# Follow-up and screening for type-2 diabetes mellitus in women with previous gestational diabetes in primary care

**DOI:** 10.1080/02813432.2023.2182632

**Published:** 2023-02-28

**Authors:** Amanda Björk Javanshiri, Susanna Calling, Sara Modig

**Affiliations:** Center for Primary Health Care Research, Department of Clinical Sciences Malmö, Lund University, Lund, Sweden

**Keywords:** Follow-up, gestational diabetes mellitus, prevention, primary health care, type 2 diabetes mellitus

## Abstract

**Objective:**

Gestational diabetes mellitus (GDM) is an established risk factor for developing type 2 diabetes mellitus (T2DM) that is possible to prevent by systematic follow-up and preventive measures. The aim of this study was to examine whether women with previous GDM were offered follow-up in primary care, according to Swedish national guidelines.

**Design:**

Retrospective review of electronic medical records.

**Setting:**

Primary care in southern Sweden, Skåne county.

**Subjects:**

Women who received a GDM diagnosis in 2018 at the Endocrinology department, Skåne University Hospital in Lund. The study population consisted of a total number of 161 patients, whereof 83 patients were included.

**Main outcome measures:**

Whether primary care offered follow-up for T2DM after GDM and if any communication took place between secondary and primary care. Furthermore, it was examined whether the quality of the follow-up was in accordance with the national guidelines.

**Results:**

Of the study population, a total of 29% (*n* = 24) had been followed-up by primary care. In 55% (*n* = 46) of the cases, there was no communication between secondary and primary care. Plasma glucose was checked in all (*n* = 20) cases where follow-up could be evaluated. Conversations about lifestyle habits took place in 70% (*n* = 14) of the cases. Weight and risk factors for cardiovascular disease were controlled in less than half (*n* = 9) of the patients. Lifestyle advice was offered in two cases and in 24% (*n* = 20) of the cases an annual check-up was planned.

**Conclusions:**

The follow-up of women with previous GDM in primary care in southern Sweden was lacking in seven out of 10 cases and showed great potential for improvement.

**Key Points**
- Gestational diabetes is an established risk factor for developing type 2 diabetes.
- Earlier research has recognized that risk reduction is possible by systematic follow-up and preventive measures, but the extent of follow-up in primary care in southern Sweden remains unknown.
- This study demonstrates a lack of follow-up according to national guidelines for women with previous gestational diabetes in primary care in southern Sweden.
- There is great potential to improve the care of these patients with relatively simple means.

## Introduction

Gestational diabetes mellitus (GDM), one of the most common pregnancy complications, is defined as hyperglycemia first diagnosed during pregnancy [1]. Type 2 diabetes mellitus (T2DM) as well as GDM prevalence are both increasing and contribute in the long-term to a considerable global health burden. Internationally, the prevalence is 9–26% [2] and in Europe between 2 and 6% of mothers are diagnosed with GDM. However, since international consensus about screening policy, testing methods and diagnostic criteria is lacking, the prevalence is difficult to assess [3]. Increasing maternal age and being overweight are believed to be associated with the rising prevalence [4]. In Region Skåne, the most southern county in Sweden, the incidence is among the highest in Sweden; 3.1% compared to 1.7% nationally. High participation in the regional screening program for GDM among pregnant women (95%) might partly explain this [5].

GDM increases the risk of several perinatal complications such as preeclampsia, macrosomia and delivery complications [5,6]. It is an established fact that GDM not only causes complications related to pregnancy but also substantially increases the risk of T2DM [4,7]. The risk increase for T2DM is 7–10 times higher compared to normoglycemic pregnancies [4,7] and the lifetime risk of developing T2DM is as high as 70% with the most rapid increase occurring during the first 10 years after giving birth [8]. Lee et al. and Linné et al. concluded that a quarter to a third of GDM patients had developed T2DM after a follow-up period of 15 years [9,10].

Since GDM is regarded as a major risk factor for future T2DM, Swedish national guidelines recommend systematic follow-up and screening of these patients postpartum [1]. This recommendation is strengthened by an intervention study – The Diabetes Prevention Program – which concluded that prevention, dietary advice and exercise, decreased the risk of T2DM by 58% [11]. The risk reduction persisted 10 years after the study was conducted, with the incidence of T2DM being 34% lower in the intervention group. In addition, it was found that a possible diabetes onset was delayed by four years in this group [12]. In Sweden, national guidelines state that primary care should be responsible for systematic follow-up of women with previous GDM that should constitute of the following: control of fasting plasma glucose and weight, support in changing unhealthy lifestyles and reviewing other risk factors for cardiovascular disease [5,13]. This is to prevent the risk of developing T2DM as well as to avoid or delay diabetes complications by early detection. These relatively simple measures should be highly prioritized since it is believed to prevent disease in this high-risk population [1,13]. However, it is unclear how often the follow-up should be conducted, although local guidelines recommend annually or every other year. In addition, a check-up should always be performed before planning a new pregnancy to avoid undiagnosed diabetes, which may pose an additional risk to the fetus [6].

The aim of this study was to examine whether women with previous GDM were offered follow-up in primary care in Region Skåne in accordance with Swedish national guidelines.

## Materials and methods

### Design and setting

This study constituted of a retrospective review of medical records, with the study population consisting of women diagnosed with previous GDM. In Region Skåne, an oral glucose tolerance test (OGTT) is offered to all pregnant women in pregnancy week 28. Fasting plasma glucose ≥7.0 or ≥10.0, two hours after OGTT (capillary sampling) is assessed as GDM and the patient is then referred to the specialist prenatal care unit at the hospital for regular check-ups during pregnancy [5]. Women with previous GDM are then offered postpartum screening one year after delivery with OGTT conducted by the Endocrinology department at one of the larger public hospitals in the region. Thereafter, they should be referred to primary care for continuous follow-up.

### Data sources and measurement

The following women were identified and included:

Women who in 2018 had a follow-up one year postpartum at the Endocrinology department, Skåne University Hospital in Lund, under the diagnosis GDM (International Classification of Diseases, ICD-10) ICD O24.4 and O24.4X. The ICD codes were then linked to a personal identification number.

The following women were excluded:

- Women who at the check-up were diagnosed with manifest diabetes mellitus.
- Women who had moved from the geographic relevant area.
- Women who were registered at private primary health care centers (PHCC) >1 month during the study period.
- Women who became pregnant anew during the study period.
- One blocked medical record that could not be accessed.

**Figure 1.**
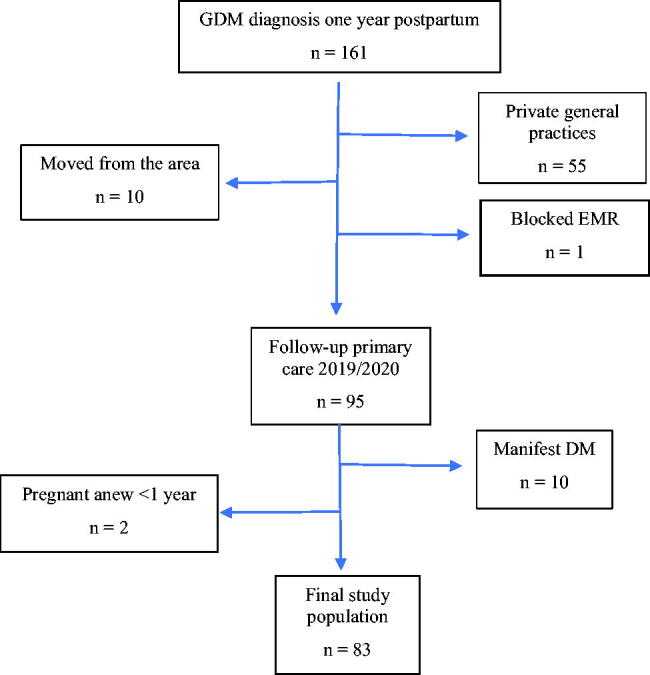
Flowchart of the study population.

A flowchart of the study population is illustrated in Figure 1. Thereafter, an electronic medical record (EMR) review was performed to see if the patients were ever offered follow-up during the time period 1 January 2019 to 31 December 2020, and if so, the extent of follow-up was also registered.

### Outcomes

During the medical records review, the following variables were sought:

- whether patients had been offered follow-up either via a doctor or a nurse specialized in diabetes,
- if the communication between secondary and primary care took place by incoming referral/copy of medical records or via the patient herself.

In addition, the quality of the follow-up and whether it was in accordance with the national guidelines were examined:

- by control of plasma glucose and weight as well as,
- conversation about unhealthy lifestyles (dietary- and exercise advice) and,
- reviewing other risk factors for cardiovascular disease (blood pressure, blood lipids and tobacco use).

Finally, it was investigated whether the patients were offered primary preventive measures:

- dietitian- or physiotherapist contact and annual check-up.

### Statistical analyses

Descriptive statistics (numbers and proportions) were used to describe the frequency and extent of follow-up.

### Ethics

Ethical approval was obtained by the Swedish Ethical Review Authority (2021-00898).

## Results

The study population consisted of a total number of 161 patients, whereof 83 patients were included. After the initial one-year postpartum follow-up at the Endocrinology department at secondary care, 24 patients (29%) were followed-up by primary care, see Table 1 for details. Of those offered follow-ups, communication with secondary care took place for all 23 patients, except one (96%). A total of 59 patients (71%) had not been offered any follow-up, even though in 12 (20%) of these cases, a referral/medical records copy had been received from the hospital. In 46 (55%) cases, there did not appear to be any communication between secondary and primary care and in all these cases follow-up defaulted. Communication with secondary care took place in 35 (42%) cases, as shown in Table 1. In five cases, the patient herself contacted the primary healthcare center, and in three of these cases, there was also other communication with secondary care through incoming referrals or medical record copies.

**Table 1.** Follow-up of women with previous GDM in primary care, *N* = 83.

Follow-up of women with previous GDM	*n* (%)
Total no. of patients being followed-up in primary care	24 (29)
Check-up by doctor only	9 (11)
Check-up by diabetes nurse only	15 (18)
Check-up by doctor and nurse	1 (1)
Check-up by unclear profession	1 (1)
Total no. of cases with communication between secondary and primary care	35 (42)
Individual referrals	17 (20)
Referrals together with medical record copies	14 (17)
Individual medical record copies	4 (5)
No. of patients who contacted primary care themselves	5 (6)

**Figure 2.**
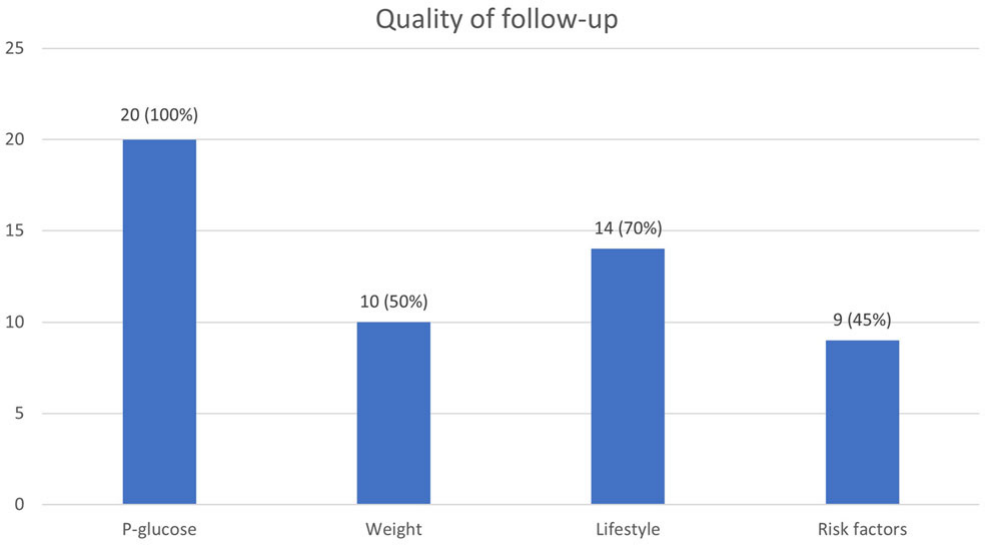
The proportion of follow-up for each parameter according to Swedish national guidelines.

In 20 out of 24 cases, the quality of the follow-up could be evaluated since in three cases an annual check-up was planned outside the study period and in one case the patient did not attend. Of the 20 patients who were followed up in primary care within the study period, plasma glucose was checked in all (100%) cases. Additional quality measures are shown in Figure 2.

Prevention was offered in two cases, who were offered to meet a dietitian. One of these was also offered prescribed physical activity (although it was the patient’s own wish). Annual check-ups were planned in 20 cases, i.e. 83% of those offered follow-up, corresponding to 24% of the total study population.

## Discussion

This study demonstrates a lack of follow-up and screening for T2DM in women with previous GDM in primary care in southern Sweden. Furthermore, the quality of the follow-up did not correspond with the national guidelines. In particular, the preventive measures that these patients can be offered in order to reduce the risk of developing T2DM failed.

The main study limitation is the modest study population, which makes the results difficult to generalize, other than possibly to similar populations in Scandinavia. The limited study time may be a source of error, as both communication with secondary care as well as follow-up might have occurred before or after the study period. In some cases, the follow-up was performed via phone instead of physical visits, which can be partly explained by primary care receiving directives to reduce physical contact, when it was possible, due to the Covid-19 pandemic in the latter part of the study follow-up. One can speculate that the preventive work may also have been downgraded to a greater extent due to the pandemic, both by healthcare staff and patients. However, the follow-up in 2019 was also insufficient, thus before the start of the pandemic. Other limitations are incomplete medical records, where it is impossible to tell whether future follow-up was planned outside of the study period, as well as if the patients were offered dietician/physiotherapist contact but declined. It is possible that some of the patients were offered further follow-up by secondary care for another reason, which in that case would explain why no referral or medical record copy was received in a few cases. To retrieve medical records, a regional consent (KVB 110-21) was needed and unfortunately this did not include private PHCC. This is a major limitation to the study since it might contribute to selection bias. Furthermore, data about baseline characteristics were lacking which could contribute to bias since it is a well-known fact that women of higher socioeconomic status is more likely to attend follow-up [14]. However, since the regional consent was obtained for patient data, it is reasonable to assume that we included all feasible patients in the study. The fact that the follow-up period extends over two years can also be considered a strength, as most studies regarding follow-up focus on the initial postpartum period a few weeks after delivery.

The study results are in line with prior research that concluded that postpartum screening is often deficient [14–16]. Postpartum screening varies widely globally, ranging from 19 to 73% of women with previous GDM being followed up after delivery. However, the definition of GDM, the follow-up time and the screening method differs broadly [15]. In a British retrospective cohort study from 2018, 58% were followed up one year postpartum, but only 40% after two years [16]. A Danish register study from 2014 also concluded that follow-up declines over time since 47% were followed up after two years, then gradually reducing to 29% after four years and barely 18% after six years [14]. The same study also concluded that attending regular primary care follow-up increased the risk of diabetes diagnosis and treatment (OR 11.8), which also indicates that non-attenders were at increased risk of undiagnosed diabetes, delayed diagnosis as well as treatment and thus increased risk of complications [14].

There is a clear association between GDM and T2DM, with the diseases sharing pathophysiology, risk factors and genetic risk alleles [8]. Regardless of the strong association between the diseases along with national guidelines urging the importance of this high-risk population being followed-up, the results indicate a lack thereof. Previous studies have tried to explain the reason behind the lack of follow-up. A review article has shown that insufficient communication between secondary and primary care can be one cause [17], which is also clear from the results in the present study. In a national UK study, general practitioners reported that they were not informed in a fifth of the cases that their patients were diagnosed with GDM and only four out of 10 had established routines on how women should be followed-up [18]. The fact that women, despite the information, are unaware of the risk of developing T2DM and often regard GDM as a transient condition, may be another reason. A US study found that 90% of women with a history of GDM were aware of the association between GDM and T2DM, but only 16% perceived that they themselves were at higher risk of developing T2DM [19]. Furthermore, qualitative studies have shown that women, as new mothers, find it difficult to prioritize their own health as well as finding the time for check-ups [17,20]. This is also suggested by the current study as the women included, despite encouragement from the hospital to contact primary care for future follow-up, only did so in a few cases. In addition, they were offered follow-up in some cases but did not attend. Poor socioeconomic status has also been demonstrated to have a negative effect on follow-up participation [14]. Although the Skåne University Hospital in Lund area corresponds to a region with a demographically very heterogeneous population, this was not further investigated. Lack of knowledge among healthcare professionals and deficient continuity are also likely to contribute [17]. The EMR review revealed that the patients’ previous GDM diagnosis was often noted, but that this did not seem to initiate any further checks. The lack of quality also implies a need for better understanding of the national guidelines for diabetes care.

Systematic, lifelong follow-up in primary care is essential to prevent the risk of developing T2DM as well as to inhibit and delay diabetic complications. Therefore, it is important to improve future care. According to our study, there is a potential for improvement regarding awareness among both caregivers and patients, communication with secondary care as well as finding ways to increase compliance with regular check-ups. A multidisciplinary provision of care involving two specialist clinics (maternity care and endocrine) as well as primary care requires a clear division of responsibilities and adequate information transfer. Perhaps there is a need for improved guidelines regarding how these patients should be managed by primary care to increase knowledge. One can assume that this group of patients are not prioritized in an overloaded primary care, where the prevention work is at risk of being downgraded. Several studies have demonstrated that the best way to improve adherence to follow-up in primary care is through a proactive reminder system, for example via phone calls/SMS/letters/personal nurse contact [17,20,21]. Combining the T2DM screening with another healthcare visit after delivery, for example at the childcare center could be a solution [15]. Such a procedure has been implemented in one of the general practices included in the study.

In 2015, the Swedish National Board of Health and Welfare (SNBHW) recommended new guidelines, introducing lowered cut-off values for GDM, since the HAPO study in 2008 found that untreated hyperglycemia, even below the threshold values for GDM, increased the risk of a negative birth outcome [13,22]. A current large national randomized prospective control study is evaluating the short- and long-term effects of the new guidelines and the consequences from a health economic perspective [23].

Our study focused on the patients that attended the postpartum follow-up one year after delivery at the Endocrinology department and were then referred to primary care. The study did not include patients followed by specialist prenatal care due to GDM during the pregnancy. Presumably, there is a loss to follow-up already between the specialist clinics. Thus, the proportion being followed up is almost certainly even lower in the population at large. Therefore, a register-based study examining this would be interesting. Further qualitative studies are needed to examine how to increase adherence to regular check-ups among women with a history of GDM.

## Conclusions

Our study demonstrates that a low proportion of women with previous GDM is followed-up in primary care in southern Sweden. In addition, the study concludes that there is great potential to improve the care of these patients with relatively simple means. Ultimately, improved care could prevent the risk of developing T2DM in women with previous GDM, which would be beneficial, not only for these women’s health and long-term outcomes but also from a health economic perspective.
